# Crystal structure of 3-bromo­acetyl-6-chloro-2*H*-1-benzo­pyran-2-one

**DOI:** 10.1107/S2056989015012955

**Published:** 2015-07-31

**Authors:** Ramanaiah Chennuru, Balaji Maddimsetti, Suman Gundlapalli, R. Ravi Chandra Babu, Sudarshan Mahapatra

**Affiliations:** a Dr Reddys Laboratory, Innovation Plaza, IPDO, Bachupally, Hyderabad 500 090, India; b GITAM University, Department of Chemistry, College of Science, Vishakapatnam, Andhrapradesh, India

**Keywords:** crystal structure, coumarin, hydrogen bonding

## Abstract

In the title compound, C_11_H_6_BrClO_3_, the benzo­pyran ring system is essentially planar, with a maximum deviation of 0.036 (2) Å for the O atom. The Cl and Br atoms are displaced by −0.0526 (8) and 0.6698 (3) Å, respectively, from the mean plane of this ring system. In the crystal, two pairs of weak C—H⋯O hydrogen bonds to the same acceptor O atom link mol­ecules into inversion dimers.

## Related literature   

For applications of coumarins, see: Kale & Patwardhan (2014 ▸); Eid *et al.* (1994 ▸); Hsieh (2015 ▸); Ballazhi *et al.* (2015 ▸); Wang (2015 ▸); Lanoot *et al.* (2002 ▸); Morris & Russell (1971 ▸); Hooper *et al.* (1982 ▸); Khalfan *et al.* (1987 ▸). For related structures, see: Munshi *et al.* (2004 ▸); Munshi & Guru Row (2006 ▸); Chopra *et al.* (2006 ▸, 2007*a* ▸,*b* ▸).
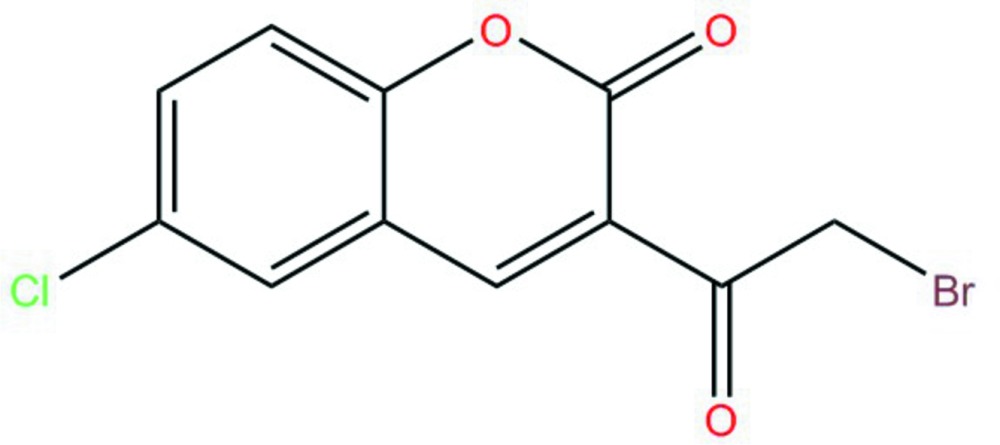


## Experimental   

### Crystal data  

C_11_H_6_BrClO_3_
*M* *_r_* = 301.51
Monoclinic, 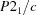

*a* = 12.5770 (2) Å
*b* = 5.7977 (1) Å
*c* = 14.8390 (3) Å
β = 94.679 (2)°
*V* = 1078.42 (3) Å^3^
*Z* = 4
Mo *K*α radiation
μ = 4.05 mm^−1^
*T* = 293 K
0.40 × 0.10 × 0.09 mm

### Data collection  

Bruker SMART CCD area-detector diffractometer
Absorption correction: multi-scan (*SADABS*; Sheldrick, 1996 ▸) *T*_min_ = 0.295, *T*_max_ = 0.712
20627 measured reflections
2113 independent reflections
1532 reflections with *I* > 2σ(*I*)
*R*_int_ = 0.031

### Refinement  

*R*[*F* ^2^ > 2σ(*F* ^2^)] = 0.030
*wR*(*F* ^2^) = 0.076
*S* = 0.95
2113 reflections
145 parameters
H-atom parameters constrained
Δρ_max_ = 0.43 e Å^−3^
Δρ_min_ = −0.60 e Å^−3^

### 

Data collection: *SMART* (Bruker, 1998 ▸); cell refinement: *SAINT* (Bruker, 1998 ▸); data reduction: *SAINT*; program(s) used to solve structure: *SHELXS97* (Sheldrick, 2008 ▸); program(s) used to refine structure: *SHELXL97* (Sheldrick, 2008 ▸); molecular graphics: *ORTEP-3* for Window (Farrugia, 2012 ▸); software used to prepare material for publication: *PLATON* (Spek, 2009 ▸).

## Supplementary Material

Crystal structure: contains datablock(s) global, I. DOI: 10.1107/S2056989015012955/lh5773sup1.cif

Structure factors: contains datablock(s) I. DOI: 10.1107/S2056989015012955/lh5773Isup2.hkl

Supporting information file. DOI: 10.1107/S2056989015012955/lh5773Isup3.cml

**Fig. 1.**
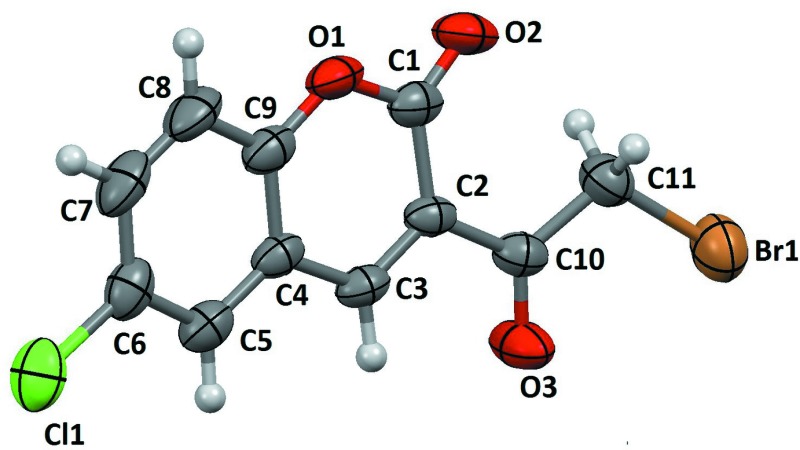
The molecular structure of the title compound with displacement ellipsoids for non-H atoms drawn at the 50% probability level.

. DOI: 10.1107/S2056989015012955/lh5773fig1.tif
The mol­ecular structure of the title compound with displacement ellipsoids for non-H atoms drawn at the 50% probability level.

**Fig. 2.**
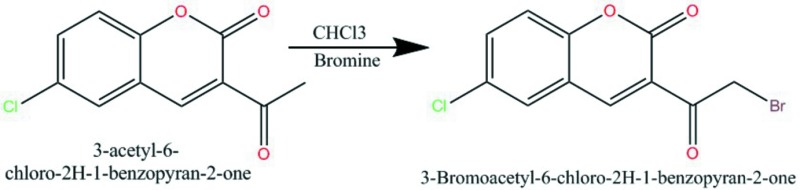
The reaction scheme.

. DOI: 10.1107/S2056989015012955/lh5773fig2.tif
The reaction scheme.

CCDC reference: 739322

Additional supporting information:  crystallographic information; 3D view; checkCIF report

## Figures and Tables

**Table 1:** Hydrogen-bond geometry (, )

*D*H*A*	*D*H	H*A*	*D* *A*	*D*H*A*
C3H3O3^i^	0.93	2.44	3.268(3)	148
C5H5O3^i^	0.93	2.54	3.337(3)	144

Symmetry code: (i) 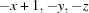.

## supplementary crystallographic information

### S1. Structural commentary

Coumarins have wide application in the pharmaceutical industry for their anti­viral activity (Kale *et al.*, 2014) and anti­microbial activity (Eid *et al.*, 1994). Recently anti­bacterial activity of the coumarin derivative chloro-chromen-2-one was studied by Lulzime *et al.*, 2015. The coumarin family can also inhibit breast cancer-mediated osteoclastogenesis and this was recently studied (Hsieh *et al.*, 2015; Ballazhi *et al.*, 2015). Further applications of coumarin derivatives for fever, inflammation and pain has been evaluated (Wang *et al.*, 2015). The well known anti­biotic Novobiocin (Lanoot *et al.*, 2002; Morris *et al.*, 1971) belongs to coumarin family. The title compound belongs to the 3-acetyl coumarin family. This coumarin family has potential application in the pharmaceutical field, dye industry and developing LASER dyes (Hooper *et al.*, 1982; Khalfan *et al.*, 1987). The crystal structure of the title coumarin derivative is reported herein.

There are two polymorphic forms of 3-acetyl coumarin reported (Munshi *et al.*, 2004; Munshi *et al.*, 2006). In both cases the structure directing inter­actions are weak C—H···O hydrogen bonds. In one form (Munshi *et al.*, 2004), a sheet-like structure is formed with two independent molecules in the asymmetric unit and in other form (Munshi *et al.*, 2006) the supra­molecular assembly is formed via inter-penetrating sheets with one molecule in the asymmetric unit and contains inversion dimer units connected through weak C—H···O inter­actions. With the substitution of bromine and chlorine (Chopra *et al.*, 2006;2007a,b) in 3-acetyl coumarin there is no significant differnce in the packing and type of weak inter­actions. In the crystal of the title compound, pairs of bifurcated –(C—H)_2_···O hydrogen bonds form inversion dimers. The molecular structure of the title compound is shown in Fig. 1.

### S2. Synthesis and crystallization

Synthesis of 3-Bromo­acetyl-6-chloro-2H-1-benzo­pyran-2-one : To a solution of 3-acetyl-6-chloro-2H-1-benzo­pyran-2-one (222mg, 1mmol) in alcohol free chloro­form (5ml), bromine (173.8 mg, 1.1 mmol) in chloro­form (2ml) was added with inter­mittent shaking and warming. The mixture was heated for fifteen minutes on a water bath, cooled and filtered. The solid was washed with ether and crystallized from glacial acetic acid to yield 3-bromo­acetyl-6-chloro-2H-1-benzo­pyran-2-one. Needle shape crystals were obtained by dissolving the title compound in glacial acetic acid and warming for a few minutes in a 10ml beaker. The beaker was covered with paraffin film with few holes in it and left till crystals appeared.

### S3. Refinement

All H atoms were positioned geometrically and refined using a riding-model approximation with C—H = 0.93 or 0.97 Å and U_iso_(H) = 1.2U_eq_(C).

### Crystal data

**Table:**

C_11_H_6_BrClO_3_	*F*(000) = 592
*M**_r_* = 301.51	*D*_x_ = 1.857 Mg m^−^^3^
Monoclinic, *P*2_1_/*c*	Mo *K*α radiation, λ = 0.71073 Å
Hall symbol: -P 2ybc	Cell parameters from 2113 reflections
*a* = 12.5770 (2) Å	θ = 3.1–26.0°
*b* = 5.7977 (1) Å	µ = 4.05 mm^−^^1^
*c* = 14.8390 (3) Å	*T* = 293 K
β = 94.679 (2)°	Needle, yellow
*V* = 1078.42 (3) Å^3^	0.40 × 0.10 × 0.09 mm
*Z* = 4	

### Data collection

**Table:**

Bruker SMART CCD area-detector diffractometer	2113 independent reflections
Radiation source: fine-focus sealed tube	1532 reflections with *I* > 2σ(*I*)
Graphite monochromator	*R*_int_ = 0.031
φ and ω scans	θ_max_ = 26.0°, θ_min_ = 3.1°
Absorption correction: multi-scan (*SADABS*; Sheldrick, 1996)	*h* = −15→15
*T*_min_ = 0.295, *T*_max_ = 0.712	*k* = −7→7
20627 measured reflections	*l* = −18→18

### Refinement

**Table:**

Refinement on *F*^2^	Primary atom site location: structure-invariant direct methods
Least-squares matrix: full	Secondary atom site location: difference Fourier map
*R*[*F*^2^ > 2σ(*F*^2^)] = 0.030	Hydrogen site location: inferred from neighbouring sites
*wR*(*F*^2^) = 0.076	H-atom parameters constrained
*S* = 0.95	*w* = 1/[σ^2^(*F*_o_^2^) + (0.0424*P*)^2^ + 0.3438*P*] where *P* = (*F*_o_^2^ + 2*F*_c_^2^)/3
2113 reflections	(Δ/σ)_max_ = 0.001
145 parameters	Δρ_max_ = 0.43 e Å^−^^3^
0 restraints	Δρ_min_ = −0.60 e Å^−^^3^

### Special details

**Table:**

Geometry. Bond distances, angles *etc*. have been calculated using the rounded fractional coordinates. All su's are estimated from the variances of the (full) variance-covariance matrix. The cell e.s.d.'s are taken into account in the estimation of distances, angles and torsion angles
Refinement. Refinement of *F*^2^ against ALL reflections. The weighted *R*-factor *wR* and goodness of fit *S* are based on *F*^2^, conventional *R*-factors *R* are based on *F*, with *F* set to zero for negative *F*^2^. The threshold expression of *F*^2^ > σ(*F*^2^) is used only for calculating *R*-factors(gt) *etc*. and is not relevant to the choice of reflections for refinement. *R*-factors based on *F*^2^ are statistically about twice as large as those based on *F*, and *R*- factors based on ALL data will be even larger.

### Fractional atomic coordinates and isotropic or equivalent isotropic displacement parameters (Å^2^)

**Table:**

	*x*	*y*	*z*	*U*_iso_*/*U*_eq_	
Br1	0.82908 (2)	0.43513 (6)	0.08551 (2)	0.0724 (1)	
Cl1	0.03785 (6)	−0.08546 (14)	0.11305 (5)	0.0667 (3)	
O1	0.36571 (16)	0.6170 (3)	0.19323 (11)	0.0522 (6)	
O2	0.52243 (17)	0.7733 (3)	0.18198 (12)	0.0592 (7)	
O3	0.62089 (15)	0.2132 (3)	0.03489 (14)	0.0665 (7)	
C1	0.4668 (2)	0.6090 (4)	0.16473 (15)	0.0450 (9)	
C2	0.4945 (2)	0.4025 (4)	0.11494 (14)	0.0380 (8)	
C3	0.4205 (2)	0.2395 (4)	0.09438 (15)	0.0396 (8)	
C4	0.3152 (2)	0.2556 (4)	0.12289 (15)	0.0403 (8)	
C5	0.2359 (2)	0.0892 (4)	0.10347 (16)	0.0453 (8)	
C6	0.1383 (2)	0.1173 (5)	0.13694 (17)	0.0507 (9)	
C7	0.1178 (3)	0.3055 (6)	0.19074 (19)	0.0624 (10)	
C8	0.1941 (3)	0.4691 (6)	0.21009 (19)	0.0616 (11)	
C9	0.2920 (2)	0.4454 (4)	0.17544 (16)	0.0476 (8)	
C10	0.6045 (2)	0.3676 (4)	0.08674 (15)	0.0422 (8)	
C11	0.6926 (2)	0.5239 (5)	0.12365 (18)	0.0546 (9)	
H3	0.43848	0.11242	0.06054	0.0475*	
H5	0.24924	−0.03874	0.06832	0.0543*	
H7	0.05146	0.32003	0.21373	0.0747*	
H8	0.18033	0.59506	0.24616	0.0741*	
H11A	0.67683	0.68051	0.10379	0.0655*	
H11B	0.69582	0.52191	0.18918	0.0655*	

### Atomic displacement parameters (Å^2^)

**Table:**

	*U*^11^	*U*^22^	*U*^33^	*U*^12^	*U*^13^	*U*^23^
Br1	0.0534 (2)	0.0854 (3)	0.0780 (2)	−0.0081 (2)	0.0030 (2)	−0.0066 (2)
Cl1	0.0485 (4)	0.0861 (6)	0.0667 (4)	0.0015 (4)	0.0129 (3)	0.0141 (4)
O1	0.0676 (13)	0.0426 (10)	0.0454 (10)	0.0208 (9)	−0.0008 (9)	−0.0103 (8)
O2	0.0855 (15)	0.0367 (10)	0.0539 (11)	0.0024 (10)	−0.0027 (10)	−0.0138 (9)
O3	0.0569 (12)	0.0673 (13)	0.0782 (13)	−0.0123 (10)	0.0231 (10)	−0.0361 (12)
C1	0.0644 (18)	0.0372 (15)	0.0318 (12)	0.0127 (13)	−0.0050 (12)	0.0010 (11)
C2	0.0543 (15)	0.0308 (13)	0.0285 (11)	0.0060 (11)	0.0008 (10)	−0.0006 (10)
C3	0.0543 (15)	0.0338 (13)	0.0313 (12)	0.0110 (12)	0.0075 (11)	−0.0007 (10)
C4	0.0516 (15)	0.0379 (14)	0.0315 (12)	0.0154 (12)	0.0049 (10)	0.0030 (10)
C5	0.0509 (15)	0.0454 (15)	0.0405 (13)	0.0126 (13)	0.0096 (11)	0.0052 (11)
C6	0.0472 (16)	0.0639 (18)	0.0411 (14)	0.0130 (13)	0.0050 (12)	0.0120 (13)
C7	0.0523 (18)	0.083 (2)	0.0535 (16)	0.0289 (17)	0.0142 (14)	0.0042 (16)
C8	0.065 (2)	0.069 (2)	0.0515 (16)	0.0325 (17)	0.0086 (14)	−0.0107 (14)
C9	0.0573 (16)	0.0468 (15)	0.0383 (12)	0.0195 (14)	0.0013 (11)	0.0005 (12)
C10	0.0558 (16)	0.0361 (13)	0.0348 (12)	−0.0014 (11)	0.0036 (11)	−0.0017 (11)
C11	0.0595 (18)	0.0532 (16)	0.0497 (15)	−0.0032 (13)	−0.0034 (13)	−0.0071 (13)

### Geometric parameters (Å, º)

**Table:**

Br1—C11	1.921 (3)	C5—C6	1.371 (4)
Cl1—C6	1.741 (3)	C6—C7	1.389 (4)
O1—C1	1.373 (3)	C7—C8	1.363 (5)
O1—C9	1.371 (3)	C8—C9	1.379 (4)
O2—C1	1.197 (3)	C10—C11	1.500 (4)
O3—C10	1.209 (3)	C3—H3	0.9300
C1—C2	1.464 (3)	C5—H5	0.9300
C2—C3	1.344 (3)	C7—H7	0.9300
C2—C10	1.492 (4)	C8—H8	0.9300
C3—C4	1.426 (4)	C11—H11A	0.9700
C4—C5	1.401 (3)	C11—H11B	0.9700
C4—C9	1.393 (3)		
			
Br1···O3	2.9589 (19)	C2···C10^viii^	3.417 (3)
Br1···H8^i^	3.1900	C2···O3^viii^	3.389 (3)
Cl1···C8^ii^	3.485 (4)	C3···O2^i^	3.343 (3)
Cl1···Cl1^iii^	3.5530 (11)	C3···O2^ii^	3.220 (3)
Cl1···H7^iv^	2.9400	C3···C10^viii^	3.518 (3)
O1···C5^v^	3.403 (3)	C3···O3^vii^	3.268 (3)
O1···O2^i^	2.992 (3)	C4···O2^i^	3.406 (3)
O2···C3^v^	3.220 (3)	C5···O3^vii^	3.337 (3)
O2···C11	2.779 (3)	C5···O1^ii^	3.403 (3)
O2···C4^vi^	3.406 (3)	C8···Cl1^v^	3.485 (4)
O2···C9^vi^	3.180 (3)	C9···O2^i^	3.180 (3)
O2···C2^vi^	3.129 (3)	C10···C2^viii^	3.417 (3)
O2···O1^vi^	2.992 (3)	C10···C3^viii^	3.518 (3)
O2···C1^vi^	2.988 (3)	C11···O2	2.779 (3)
O2···C3^vi^	3.343 (3)	C1···H11B	2.9200
O3···Br1	2.9589 (19)	C1···H11A	2.8900
O3···C5^vii^	3.337 (3)	H3···O2^ii^	2.8100
O3···C1^viii^	3.244 (3)	H3···O3	2.4300
O3···C2^viii^	3.389 (3)	H3···H5	2.5500
O3···C3^vii^	3.268 (3)	H3···O3^vii^	2.4400
O2···H11A	2.4000	H5···H3	2.5500
O2···H11B	2.6200	H5···O3^vii^	2.5400
O2···H3^v^	2.8100	H7···Cl1^ix^	2.9400
O3···H3	2.4300	H8···Br1^vi^	3.1900
O3···H3^vii^	2.4400	H11A···O2	2.4000
O3···H5^vii^	2.5400	H11A···C1	2.8900
C1···O3^viii^	3.244 (3)	H11B···O2	2.6200
C1···O2^i^	2.988 (3)	H11B···C1	2.9200
C2···O2^i^	3.129 (3)		
			
C1—O1—C9	123.02 (19)	C4—C9—C8	121.4 (2)
O1—C1—O2	116.6 (2)	O3—C10—C2	119.3 (2)
O1—C1—C2	116.6 (2)	O3—C10—C11	121.4 (2)
O2—C1—C2	126.9 (2)	C2—C10—C11	119.4 (2)
C1—C2—C3	120.1 (2)	Br1—C11—C10	112.44 (18)
C1—C2—C10	121.1 (2)	C2—C3—H3	119.00
C3—C2—C10	118.8 (2)	C4—C3—H3	119.00
C2—C3—C4	122.0 (2)	C4—C5—H5	120.00
C3—C4—C5	123.8 (2)	C6—C5—H5	120.00
C3—C4—C9	117.4 (2)	C6—C7—H7	120.00
C5—C4—C9	118.8 (2)	C8—C7—H7	120.00
C4—C5—C6	119.2 (2)	C7—C8—H8	120.00
Cl1—C6—C5	120.2 (2)	C9—C8—H8	120.00
Cl1—C6—C7	118.8 (2)	Br1—C11—H11A	109.00
C5—C6—C7	121.0 (3)	Br1—C11—H11B	109.00
C6—C7—C8	120.5 (3)	C10—C11—H11A	109.00
C7—C8—C9	119.1 (3)	C10—C11—H11B	109.00
O1—C9—C4	120.8 (2)	H11A—C11—H11B	108.00
O1—C9—C8	117.8 (2)		
			
C9—O1—C1—O2	177.6 (2)	C3—C4—C5—C6	177.6 (2)
C9—O1—C1—C2	−1.3 (3)	C9—C4—C5—C6	−0.3 (3)
C1—O1—C9—C4	−3.0 (3)	C3—C4—C9—O1	4.5 (3)
C1—O1—C9—C8	177.9 (2)	C3—C4—C9—C8	−176.5 (2)
O1—C1—C2—C3	4.1 (3)	C5—C4—C9—O1	−177.5 (2)
O1—C1—C2—C10	−175.44 (19)	C5—C4—C9—C8	1.6 (4)
O2—C1—C2—C3	−174.6 (2)	C4—C5—C6—Cl1	179.43 (19)
O2—C1—C2—C10	5.9 (4)	C4—C5—C6—C7	−1.1 (4)
C1—C2—C3—C4	−2.7 (3)	Cl1—C6—C7—C8	−179.3 (2)
C10—C2—C3—C4	176.9 (2)	C5—C6—C7—C8	1.2 (4)
C1—C2—C10—O3	−169.4 (2)	C6—C7—C8—C9	0.1 (4)
C1—C2—C10—C11	10.9 (3)	C7—C8—C9—O1	177.6 (3)
C3—C2—C10—O3	11.0 (3)	C7—C8—C9—C4	−1.4 (4)
C3—C2—C10—C11	−168.7 (2)	O3—C10—C11—Br1	−3.9 (3)
C2—C3—C4—C5	−179.6 (2)	C2—C10—C11—Br1	175.83 (17)
C2—C3—C4—C9	−1.6 (3)		

Symmetry codes: (i) −*x*+1, *y*−1/2, −*z*+1/2; (ii) *x*, *y*−1, *z*; (iii) −*x*, −*y*, −*z*; (iv) −*x*, *y*−1/2, −*z*+1/2; (v) *x*, *y*+1, *z*; (vi) −*x*+1, *y*+1/2, −*z*+1/2; (vii) −*x*+1, −*y*, −*z*; (viii) −*x*+1, −*y*+1, −*z*; (ix) −*x*, *y*+1/2, −*z*+1/2.

### Hydrogen-bond geometry (Å, º)

**Table:**

*D*—H···*A*	*D*—H	H···*A*	*D*···*A*	*D*—H···*A*
C3—H3···O3^vii^	0.9300	2.4400	3.268 (3)	148.00
C5—H5···O3^vii^	0.9300	2.5400	3.337 (3)	144.00

Symmetry code: (vii) −*x*+1, −*y*, −*z*.

## References

[bb12] Ballazhi, L., Popovski, E., Jashari, A., Imeri, F., Ibrahimi, I., Mikhova, B. & Mladenovska, K. (2015). *Acta Pharm.* **65**, 53–63. 10.1515/acph-2015-0002 25781704

[bb1] Bruker (1998). *SMART* and *SAINT*. Bruker AXS Inc, Madison, Wisconsin, USA.

[bb2] Chopra, D., Venugopala, K. N. & Rao, G. K. (2007*a*). *Acta Cryst.* E**63**, o4872.

[bb3] Chopra, D., Venugopala, K. N., Rao, G. K. & Guru Row, T. N. (2007*b*). *Acta Cryst.* E**63**, o2826.

[bb4] Chopra, D., Venugopal, K. N., Jayashree, B. S. & Row, T. N. G. (2006). *Acta Cryst.* E**62**, o2310–o2312.

[bb5] Eid, A. I., Ragab, F. A., El-Ansary, S. L., El-Gazayerly, S. M. & Mourad, F. E. (1994). *Arch. Pharm. Pharm. Med. Chem.* **327**, 211–213. 10.1002/ardp.19943270404 8204021

[bb6] Farrugia, L. J. (2012). *J. Appl. Cryst.* **45**, 849–854.

[bb7] Hooper, D. C., Wolfson, J. S., McHugh, G. L., Winters, M. B. & Swartz, M. N. (1982). *Antimicrob. Agents Chemother.* **22**, 662–671. 10.1128/aac.22.4.662 PMC183811 6295263

[bb8] Hsieh, C. (2015). *Int. J. Oncol.* **46**, 2, 555–562. 10.3892/ijo.2014.2769 25421824

[bb9] Kale, M. & Patwardhan, K. (2014). *Curr. Pharm. Res.* **4**, 1150-1158.

[bb10] Khalfan, H., Abuknesha, R., Rond-Weaver, M., Price, R. G. & Robinson, R. (1987). *Chem. Abstr.* **106**, 63932.

[bb11] Lanoot, B., Vancanneyt, M., Cleenwerck, I., Wang, L., Li, W., Liu, Z. & Swings, J. (2002). *Int. J. Syst. Evol. Microbiol.* **52**, 823–829. 10.1099/00207713-52-3-823 12054245

[bb13] Morris, A. & Russell, A. D. (1971). *Prog. Med. Chem.* **8**, 39–59. 10.1016/s0079-6468(08)70127-9 4254750

[bb14] Munshi, P. & Guru Row, T. N. (2006). *Cryst. Growth Des.* **6**, 708–718.

[bb15] Munshi, P., Venugopala, K. N., Jayashree, B. S. & Guru Row, T. N. (2004). *Cryst. Growth Des.* **4**, 1105–1107.

[bb16] Sheldrick, G. M. (1996). *SADABS*. University of Göttingen, Germany.

[bb17] Sheldrick, G. M. (2008). *Acta Cryst.* A**64**, 112–122. 10.1107/S0108767307043930 18156677

[bb18] Spek, A. L. (2009). *Acta Cryst.* D**65**, 148–155. 10.1107/S090744490804362X PMC2631630 19171970

[bb19] Wang, R. (2015). CN Patent CN 104557831 A.

