# Supplementary figures and images for: Crystal structure of 3-bromo­acetyl-6-chloro-2H-1-benzo­pyran-2-one
Source: Acta Crystallogr E Crystallogr Commun. 2015 Jul 31;71(Pt 8):o615–6. doi: 10.1107/S2056989015012955 (PMC4571426; doi:10.1107/S2056989015012955)

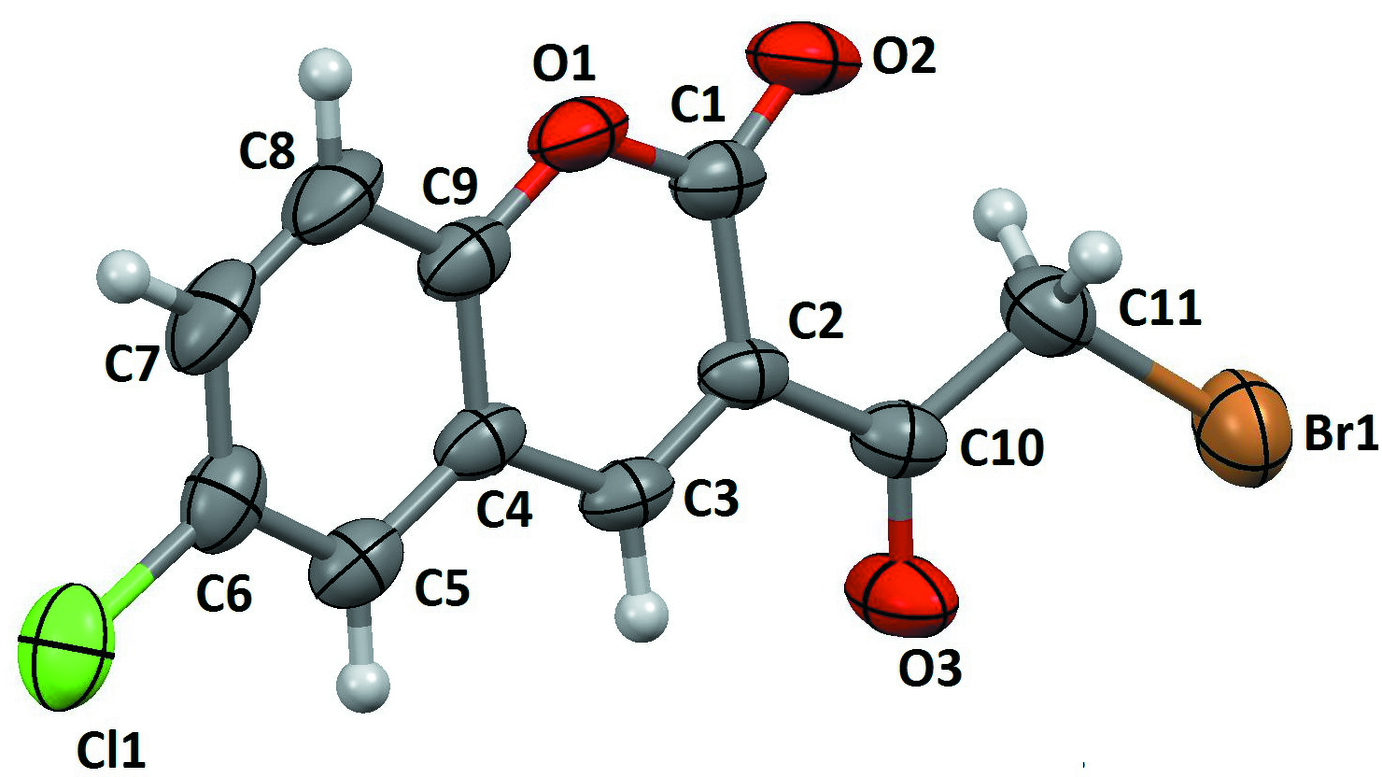

Supplement: Supplementary file 4 [file e-71-0o615-fig1.tif]

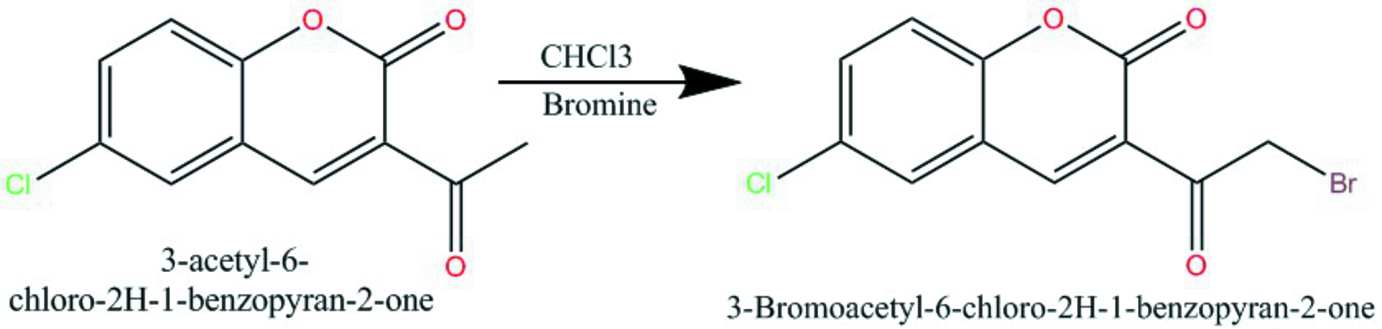

Supplement: Supplementary file 5 [file e-71-0o615-fig2.tif]
